
JOURNAL INFORMATION
==============================
NLM Title Abbreviation: ScientificWorldJournal
Journal ID: ScientificWorldJournal
NLM Journal ID: 101131163
Journal ID: TSWJ

The Scientific World Journal

ISSN: 2356-6140
EISSN: 1537-744X
Publisher: Wiley

ARTICLE INFORMATION
==============================
PMCID: PMC6084243
DOI: 10.1100/tsw.2001.418
Article ID: 728656
Article version: 1
Subjects: Extended Abstract

Pharmacology and Desensitisation of the Human RAMP3 and Rat Calcitonin Receptor-Like Receptor Combination

Hay D. L.
Choksi T.
Smith D. M.
Department of Metabolic Medicine, ICSM, London, W12 0NN, UK
Publication date: 2001
Electronic publication date: 2001 Oct 30
Volume: 1
First page: 42
Last page: 42
Copyright: Copyright © 2001 D. L. Hay et al.
Copyright year: 2001
License: This is an open access article distributed under the Creative Commons Attribution License, which permits unrestricted use, distribution, and reproduction in any medium, provided the original work is properly cited.
License URL: https://creativecommons.org/licenses/by/3.0/

==============================
4th International Meeting on Calcitonin Gene-Related Peptide (CGRP)
*TheScientificWorld* (2001) 1(S1), 42
ISSN 1532-2246; DOI 10.1100/tsw.2001.418

### PHARMACOLOGY AND DESENSITISATION OF THE HUMAN RAMP3 AND RAT CALCITONIN RECEPTOR-LIKE RECEPTOR COMBINATION

D.L. Hay, T. Choksi, and D.M. Smith
Department of Metabolic Medicine, ICSM, London, W12 0NN, U.K.

Adrenomedullin (ADM) is a multifunctional regulatory peptide with its effects mediated by specific receptors. Here we examined the characteristics of the ratCRLR/humanRAMP3 combination stably expressed in HEK 293 cells with respect to binding of calcitonin family peptides and desensitisation of the ADM cAMP response following pre-exposure to ADM or other peptides. HEK 293 cells were stably transfected with rat CRLR and human RAMP3. Northern blot analysis of RNA extracted from these cells showed high expression of CRLR and RAMP3 and barely detectable levels of RAMPs 1 and 2. $^{125}$I-ratADM binding to RAMP3/CRLR was competed for by unlabelled rat ADM with an $IC_{50}$ of 9 nM. CGRP and other calcitonin family members competed with $^{125}$I-ratADM more weakly with the following $IC_{50}$ values; α-CGRP 168 nM, β-CGRP 69 nM, $CGRP_{8-37}$ 61 nM, cys(ACM2,7)CGRP 270 nM, human $ADM_{22-52}$ 220 nM, rat amylin 117 nM, rat calcitonin >1000 nM. Both ADM and CGRP caused elevation of cAMP with ADM being the most potent ($EC_{50}$ 1.5 ± 1.3 nM vs. 18.0 ± 1.1 nM, n = 3). A 2-h preincubation of these cells with 100 nM ADM resulted in a 93 ± 1% (n = 3) reduction in the subsequent cAMP response to 100 nM ADM. A similar effect was seen with $ADM_{13-50}$ but α-CGRP was less effective at this concentration. The desensitisation caused by ADM was not mimicked by 1 mM dibutryl cAMP nor inhibited by pretreatment with the protein kinase A inhibitor H-89 (100 to 500 nM). Thus the ratCRLR/humanRAMP3 combination forms a receptor with higher affinity for ADM than CGRP which is potently desensitised by ADM. This desensitisation was not due to the activation of PKA.
